# The inhibition effects of *Lentilactobacillus buchneri*-derived membrane vesicles on AGS and HT-29 cancer cells by inducing cell apoptosis

**DOI:** 10.1038/s41598-024-53773-y

**Published:** 2024-02-07

**Authors:** Adel Abedi, Farzaneh Tafvizi, Parvaneh Jafari, Neda Akbari

**Affiliations:** 1 Microbiology Department, Faculty of Science, Arak Branch, Islamic Azad University, Arak, Iran; 2 Department of Biology, Parand Branch, Islamic Azad University, Parand, Iran

**Keywords:** Membrane vesicles, *Lentilactobacillus buchneri*, Gastric cancer, Colon cancer, Apoptosis, Scratch test, Cancer, Cell biology

## Abstract

In recent years, probiotics and their derivatives have been recognized as important therapeutic agents in the fight against cancer. Therefore, this study aimed to investigate the anticancer effects of membrane vesicles (MVs) from *Lentilactobacillus buchneri* strain HBUM07105 probiotic isolated from conventional and unprocessed yogurt in Arak province, Iran, against gastric and colon cancer cell lines. The MVs were prepared from the cell-free supernatant (CFS) of *L. buchneri* and characterized using field-emission scanning electron microscopy (FE-SEM) and transmission electron microscopy (TEM) and SPS-PAGE techniques. The anticancer activity of MVs was evaluated using MTT, flow cytometry, qRT-PCR techniques, and a scratch assay. The study investigated the anti-adenocarcinoma effect of MVs isolated from *L. buchneri* on a human gastric adenocarcinoma cell line (AGS) and a human colorectal adenocarcinoma cell line (HT-29) at 24, 48, and 72-h time intervals. The results demonstrated that all prepared concentrations (12.5, 25, 50, 100, and 200 µg/mL) of MVs reduced the viability of both types of human adenocarcinoma cells after 24, 48, and 72 h of treatment. The analysis of the apoptosis results revealed that the percentage of AGS and HT-29 cancer cells in the early and late stages of apoptosis was significantly higher after 24, 48, and 72 h of treatment compared to the untreated cancer cells. After treating both AGS and HT-29 cells with the MVs, the cells were arrested in the G0/G1 phase. These microvesicles demonstrate apoptotic activity by increasing the expression of pro-apoptotic genes (*BAX*,* CASP3*, and *CASP9*). According to the scratch test, MVs can significantly decrease the migration of HT-29 and AGS cancer cells after 24, 48, and 72 h of incubation compared to the control groups. The MVs of *L. buchneri* can also be considered a potential option for inhibiting cancer cell activities.

## Introduction

Probiotic bacteria are live microorganisms, typically bacteria or yeast, that provide health benefits to the host when consumed in adequate amounts. These microorganisms are often associated with promoting a healthy balance of gut microbiota, aiding in digestion, supporting the immune system, and potentially providing other positive effects on overall well-being. Common probiotic bacteria include strains of *Lactobacillus, Bifidobacterium,* and *Saccharomyces*. Probiotics work by positively influencing the microbial balance in the digestive system^1–3^.

They may enhance the diversity and abundance of beneficial bacteria in the gut, leading to various health effects. Probiotic bacteria offer several potential benefits, such as improving digestion, producing certain vitamins, facilitating nutrient absorption, supporting the immune system, and promoting a balanced inflammatory response. Probiotics are commonly found in fermented foods such as yogurt, kefir, and in supplement form^4,5^.

However, it is essential to note that the specific benefits of probiotics can vary depending on the probiotic strain, dosage, and individual health conditions. Probiotic bacteria are also being studied for their potential impact on mental health. Additionally, probiotics are utilized in various clinical settings, such as preventing antibiotic-associated diarrhea, managing irritable bowel syndrome (IBS), and addressing specific gastrointestinal disorders. Probiotics are believed to provide additional therapeutic benefits, including anti-cholesterol activity, relief of symptoms for lactose intolerance, stimulation of the immune response, antimicrobial activity, anti-hypertensive action, as well as anticarcinogenic, antimutagenic, and anti-colorectal cancer properties. They have been shown to reduce human disease, particularly gastrointestinal infections caused by compromised or inappropriate gut microbiota^6–13^.

A few studies have focused on the impact of yogurt cultures on the metabolism of the gastrointestinal tract, and these effects have been scientifically validated. The by-product of milk fermentation by *Lactobacillus delbrueckii* subsp. bulgaricus and *Streptococcus thermophilus*. Yogurt has long been recognized for its ability to improve human health. Yogurt bacteria have been shown to influence intestinal transit time, improve lactose digestion in individuals with lactose intolerance, and stimulate the intestinal immune system. Research has also been conducted on the immunological and genetic basis of the immunostimulatory properties of yogurt starters^14^.

One of the most important strategies for human therapeutic options may be the recent developments in dietary fortification with substances that can protect against immune system problems and reduce the risk of disease^15^. The emergence of functional foods and nutraceuticals highlights the need to reduce reliance on drugs and increase the routine consumption of fermented foods. Probiotics can be beneficial to human health when taken regularly and in sufficient amounts. In particular, probiotics and lactic acid bacteria (LAB) have been suggested to function as nutraceuticals because they have no negative health effects and can also boost the immune system, thereby increasing its resistance to a variety of disease states^16^.

The gastrointestinal tract is maintained in homeostasis by a healthy balance of bacterial populations. Therefore, a variety of circumstances, including poor nutrition, stress, gastrointestinal problems, obesity, or drug use, can lead to disturbances in intestinal homeostasis. An imbalance in the digestive tract can lead to pro-inflammatory immune function and the onset of disease, including cancer. Both local gastrointestinal malignancies and tumors located in distant parts of the body may have their origins in intestinal dysbiosis^17,18^. Numerous disorders might be prevented and treated by including LAB with high probiotic capacity in starter cultures for dairy products, which are an important part of the average person's diet^19^. In order to produce high quality, nutritious and healthy dairy products, it is necessary to identify various natural resources from which suitable LAB with beneficial probiotic, functional and technical characteristics can be isolated^20^. Non-pathogenic lactic acid producing bacteria (LAB), called *Lactobacillus* species, have probiotic properties and are scientifically suitable for industrial operations^21^. In addition, *Lactobacillus* have become a mucosal delivery platform capable of replacing others such as nanoparticles, liposomes, microspheres, immunomodulating complexes, and inactivated pathogens due to their ability to survive in the gastrointestinal tract and their attachment to the intestine^22–24^.

Archaea, bacteria, and eukaryotes all employ extracellular vesicles as a means of intracellular and extracellular interaction^25,26^. Extracellular vesicles are bilayer membrane sacs with a spherical shape. One of the ways that probiotics and the immune system communicate is through the production of membrane vesicles (MVs)^27^. MVs have a diameter ranging from 100 nm to 1 µm and are created by the straight outward budding of the plasma membrane. The capacity of MVs to encapsulate active cargo, such as proteins, DNA, RNA, and lipids, makes them special. The protein content in MVs can be 100 times higher than in cell lysate. MVs are reported to be continuously shed by bacteria, but the conditions that modulate their formation remain a mystery. Little is currently known about their production and cargo^28^.

The production of MVs by the *Lactobacillus* species *L. rhamnosus* (JB-1) and *L. plantarum* has been reported^29,30^. It has been suggested that the communication between probiotic intestinal bacteria and their mammalian hosts involves immune control by LAB MVs^27^. The significant effects of whole-cell components and supernatants of probiotic lactobacilli in preventing, inhibiting, and treating various cancers, such as lung, colon, breast, colorectal, stomach, and others, are also taken into consideration^31,32^. However, to the best of our current knowledge, there have been no studies on the anticancer properties of MVs isolated from probiotic *Lentilactobacillus buchneri.* This study aimed to investigate the anticancer and antioxidant effects of *L. buchneri* strain HBUM07105 MVs isolated from conventional and unprocessed yogurt from Arak province in Iran on gastric and colon cancer cell lines.

## Material and methods

### Bacterial strain isolation and characterization

*Lentilactobacillus buchneri* strain HBUM07105 used in this study was isolated from traditional yogurt from Arak province, Iran. It was identified by 16S rRNA sequencing and registered in GenBank under the accession number *Lentilactobacillus* sp. strain T2 (0P218100) in our previous study^33^. The safety and effectiveness of the strain were assessed according to the WHO guidelines for the evaluation of probiotic bacteria in foods. The isolated probiotic bacteria were subsequently grown on selective media (de Man Rogosa and SHARP or MRS) and incubated at 36 °C with 1% carbon dioxide.

### Preparation of MVs

The bacteria strain was grown on MRS broth and incubated at 37 °C for 48 h in a microaerophilic atmosphere (5% CO_2_). Optical density at 600 nm was used to detect bacterial growth. The incubated culture (48 h) was centrifuged at 13,000 rpm for 10 min to obtain crude cell-free supernatant (CFS). CFS was filtered through a 0.22 μm nitrocellulose membrane. The purification of membrane vesicles was performed in accordance with the previously described method with some modifications. To prepare MVs from CFS, a vacuum ultracentrifuge below 20 mbar was used (Sigma 3-30K). For this purpose, 5 mL of CFS was poured into sterile polyallomer tubes and centrifuged at 100,000×*g* for 1 h at 4 °C. The supernatant was then completely discarded, and 1 mL of sterile sodium phosphate buffer was introduced into centrifugal polyallomer tubes and vortexed for 5 min. Finally, the final volume reached 50 mL by adding phosphate buffer and was divided into 1.5 mL microtubes and stored at − 60 °C. It should be noted that the nanodrop should confirm the presence of proteins at a wavelength of 280 nm^34^.

### MVs characterization

Transmission electron microscopy (TEM) (Leo 906, Zeiss100 KV model, Germany) and Field-emission scanning electron microscopy (TESCAN MIRA3) was used to confirm the ideal preparation of the extracellular vesicles. SDS-polyacrylamide gel using a Tris/HCL buffer system was used to determine the molecular weight of the purified molecules. Membrane vesicle (MVs) samples were heated in a buffer containing 0.0625 molar TRIS-HCL, 2% SDS, 10% glycerol, and 0.001% bromophenol blue for 5 min at 100 °C. Electrophoresis was then performed on a 13% polyacrylamide gel and standard markers were applied to determine the molecular weight. Finally, the protein concentration was measured by the Bradford method to exclude nucleotide contaminants and obtain the pure protein concentration^35^.

### Cell culture

The AGS cell line, derived from human gastric adenocarcinoma, and the HT29 cell line, derived from human colorectal adenocarcinoma, the HFF cell line, derived from human foreskin fibroblasts (as normal cells) were obtained from the Pasteur Institute in Iran. All cell lines were utilized in the cytotoxicity assessment of the MVs derived from *L. buchneri*. The cell lines were developed in a liquid medium consisting of RPMI1640 and DMEM (Gibco, Carlsbad, USA), which was further enriched with 10% Fetal Bovine Serum (Gibco, Carlsbad, USA), 100 μ/mL penicillin (Gibco, Carlsbad, USA), and 100 μg/mL streptomycin (Gibco, Carlsbad, USA). The cells were cultivated at 37 °C in a humid environment containing 5% CO_2_^33^.

### MTT assay

The cells gathered by trypsinization were washed with phosphate buffer saline (PBS, Sigma, St. Louis, USA) and afterwards seeded in 96-well plates at a concentration of 1 × 10^4^ cells per well. The cells were then incubated at 37 °C under a gas mixture of 5% CO_2_ and 95% air for a period of 24 h. The cells were subjected to various doses of MVs, specifically 12.5, 25, 50, 100, and 200 µg/mL. The concentrations were performed in triplicate. The quantification of the cytotoxicity of MVs was performed by assessing the capacity of viable cells to convert the yellow dye 3-4,5-dimethyl-2-thiazolyl-2,5-diphenyl-2H-terazolium bromide (MTT) into a blue formazan product. The assessment of incubation time was conducted at intervals of 24, 48, and 72 h. Following a 48-h incubation period, the culture medium in each well was substituted with a solution of MTT (5 mg/mL in phosphate-buffered saline). The plates were subsequently incubated for a duration of 4 h under conditions of 5% CO_2_ and 95% air at a temperature of 37 °C. The MTT reagent was eliminated, and the resultant formazan crystals generated by live cells were solubilized in dimethyl sulfoxide (DMSO). The measurement of absorbance was thereafter performed using an ELISA reader set at a wavelength of 570 nm. The calculation of the proportion of growth inhibition was performed employing the following the following equation:

$$\mathrm{Viability \;of \;cells }=\mathrm{ Sample \;OD}/\mathrm{Control \;OD }\times 100$$

The impact of MVs was quantified by the utilization of IC_50_ values. The IC_50_ value, which represents the concentration of the compound at which the percentage inhibition reaches 50%, was determined as the average value derived from a minimum of two separate tests^36^.

### Apoptosis assay

The assessment of the stimulation of apoptosis was conducted with the Annexin V-FITC kit (eBioscience, Affymetrix, USA) in accordance with the guidelines provided by the manufacturer. The AGS and HT-29 cell lines were seeded at a density of 1 × 10^5^ cells per well in 6-well plates. The cells were then treated with the half-maximal inhibitory concentration (IC_50_) values of MVs and incubated for 24, 48, and 72 h. The flow cytometry apparatus (Biocompare, USA) was utilized to do the cellular analysis^36^.

### Cell cycle assay

The AGS and HT29 cell lines were cultured in 6-well plates at a density of 1 × 10^6^ cells per well and incubated in culture media for a duration of 24 h. After that, the cells were subjected to treatment with IC_50_ concentrations of MVs and subsequently incubated for durations of 24, 48, and 72 h. Following the designated incubation periods, the cells underwent a single wash with PBS. Subsequently, 50 μL of PBS was introduced to the cells and subjected to vortexing. Subsequently, a fixative solution consisting of 1 mL of cold 70% ethanol was added to the samples, followed by vortexing. To prevent clumping, the cells were agitated during the fixing period. The cells were subjected to a refrigeration period of 2 h, followed by centrifugation and a single wash with PBS. The PBS was gradually eliminated, followed by the addition of 1 mL of a solution containing the PI master mix (comprising 40 μL PI, 10 μL RNase, and 950 μL PBS) to the cellular samples. Subsequently, the cells were subjected to a 30-min incubation period at ambient temperature and subsequently assessed by flow cytometry. The data underwent analysis using the FlowJo program^33^.

### qRT-PCR assay

The evaluation of mRNA expression level of *BAX, BCL2, CASP3, CASP9*, *CCND1*, *MMP2* and *VEGF* in cell lines treated with IC_50_ concentration of MVs was performed using qRT-PCR (Bioneer, Daejeon, Korea). Initially, the extraction of total RNA was performed on both cancer cell lines that were subjected to treatment and those that were left untreated. This was accomplished using an RNA extraction kit including TRIzol reagent, following the manufacturer's instructions (Qiagen, Valencia, CA, USA). The quantity of the collected total RNA was determined using a photonanometer manufactured by IMPLEN GmbH in München, Germany. The complementary DNA (cDNA) was generated with the Revert Aid First Strand cDNA Synthesis Kit, manufactured by Fermentas in Vilnius, Lithuania. In order to carry out the procedure, a mixture for the reaction was organized. The reaction mixture consisted of 5 μL of reaction buffer (5×), 2 μL of deoxynucleotide triphosphate mixture (10 mM), 0.5 μL of the oligo dT primer, 0.5 μL of a random hexamer primer, 1 μg of the extracted RNA, 1 μL of RNase enzyme inhibitor (20 units/microliter), 1 μL of reverse transcriptase enzyme, and double-distilled water (up to a final volume of 20 μL). The temperature program was configured in the following manner: the experimental conditions consisted of four temperature intervals: 25 °C for 5 min, 42 °C for 60 min, 70 °C for 5 min, and 4 °C for 5 min. The primer sequences utilized for amplifying the target genes were documented in a prior study conducted by our research team. The qRT-PCR experiment was carried out using a Light Cycler (Bioneer, Daejeon, Korea) and followed the temperature program outlined below. The experimental conditions consisted of subjecting the sample to a temperature of 95 °C for a duration of 1 min, followed by a further exposure to the same temperature of 95 °C for a period of 15 s. Finally, the sample was subjected to a temperature of 60 °C for a duration of 1 min. The relative gene expression was determined using the ΔΔCt technique, assuming a PCR efficiency of 100%^33^.

### Scratch test to detect cell migration ability

HT-29 and AGS cells were plated into a six-well plate and cultured for 24 h to reach 80–90% confluence. A straight line perpendicular to the bottom of the plate was scratched with a sterile 200 μL pipette tip, the dropped cells were washed with PBS, the culture medium was replaced with fresh medium, and the scratch was photographed using an inverted microscope connected to a real-time imaging system. Cells in culture received IC_50_ of MVs. After treatment, the treated group was kept in the incubator for 24, 48 and 72 h before being photographed again. The scratch gap area of each set of cells at 0 and 24, 48, and 72 h was used as the basis for the relative amount of cell migration, which was calculated using ImageJ software. The formula is: relative cell migration activity (%) = (Area treatment group − 0 h − Area treatment group − 24 h)/Area treatment group − 0 h × 100%^37^.

### Statistical analysis

The data were presented as the mean value accompanied by the standard deviation (SD). The statistical disparities in the measurements were examined using Student's t-test and/or one-way univariate analysis of variance (ANOVA). A statistically significant difference, denoted by an asterisk (*), was defined as having a p value less than 0.05. The statistical study was conducted using GraphPad Prism software, specifically version 8.0.2.

### Informed consent

All authors consent to the publication of this study.

## Results

### *L. buchneri* MVs characterization

**Figure 1:**
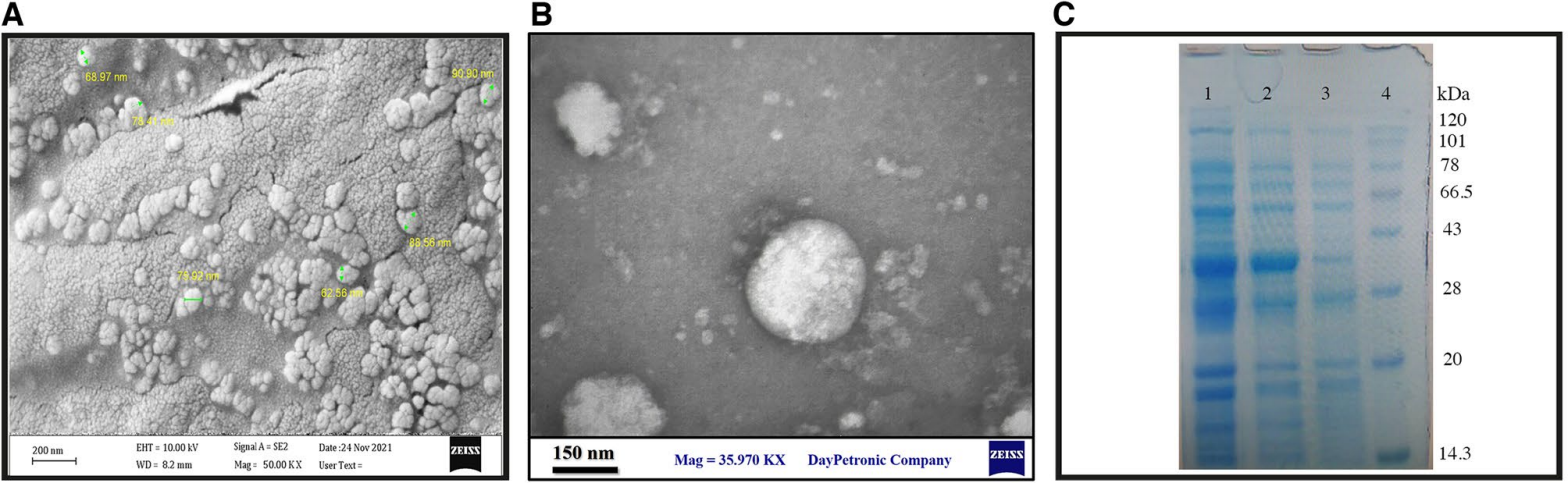
Conformation of *L. buchneri* membrane vesicles (MVs). (**A**) FE-SEM and (**B**) TEM micrographs of bacterial MVs. (**C**) SDS-PAGE of whole cell extract of *L. buchneri* (1), MVs of *L. buchneri* (2), and cell free supernatant of *L. buchneri* (CFS) (3), molecular weight Protein Marker (KDa) (4).

The morphology of MVs was spherical and concreted as well as smaller than 200 nm (Fig. 1A,B). SDS-PAGE profile was used to qualitatively analyze the proteins of MVs and supernatant isolated from *L. buchneri*. Based on the obtained results, a large percentage of proteins observed in the gel are common between cell extract, supernatant, and MVs, and it can be assumed that most of these proteins are cytoplasmic proteins cell wall related proteins and secretory proteins (Fig. 1C). The Bradford assay of passive protein concentration in MVs was 822.77 μg/mL at OD = 0.822 with R2 = 0.99. In this study, we defined the enormous protein content of MVs isolated from *L. buchneri* by SDS-PAGE technique and reported that the protein content of MVs is more than the supernatant protein content (Fig. 1C).

### Cell viability percentage

**Figure 2:**
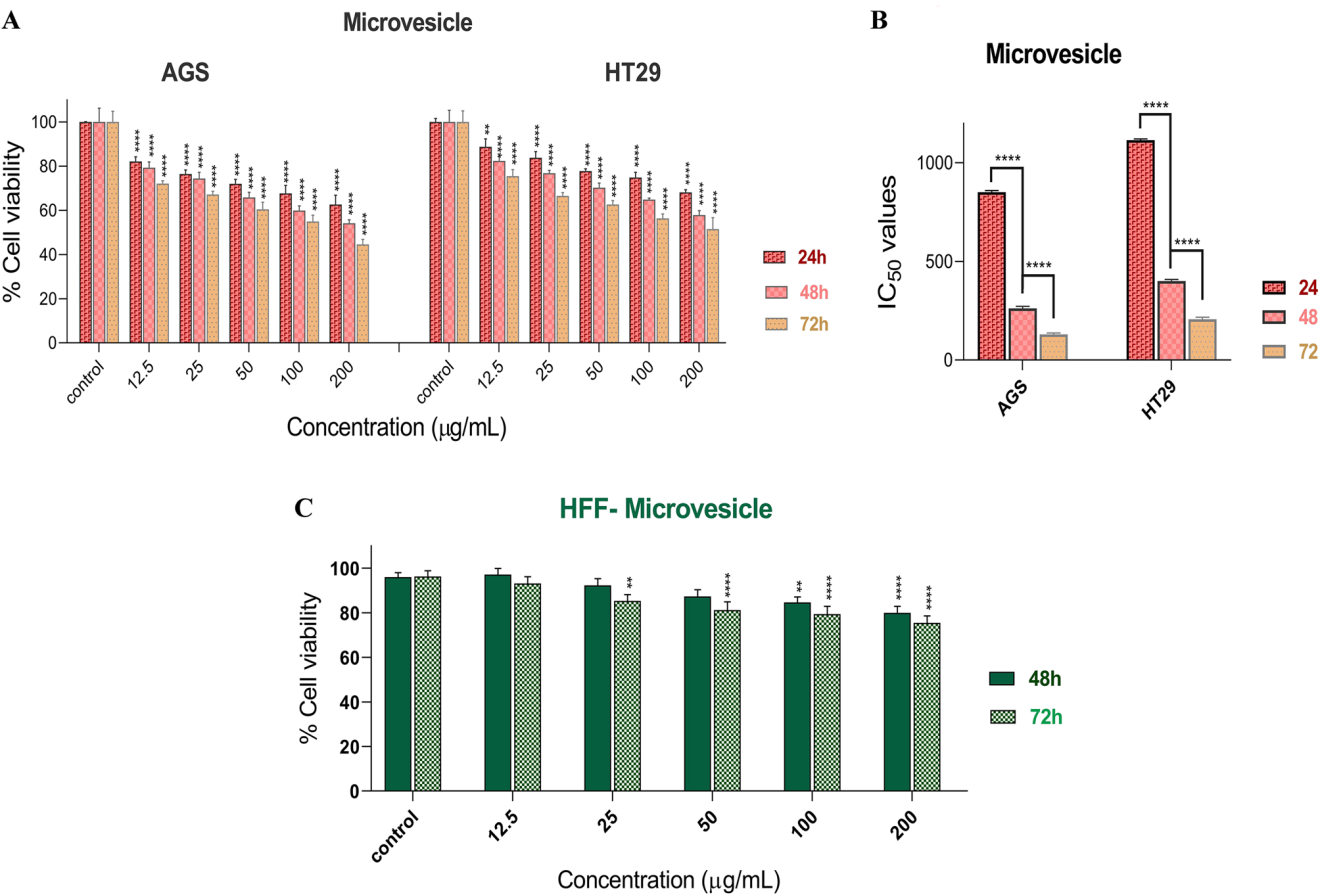
(**A**) Cell viability of AGS and HT-29 cancer cells following treatment with *L. buchneri* MVs after 24, 48, and 72 h (results are reported as viability in comparison with control group (P < 0.01**, P < 0.0001****). (**B**) IC_50_ values of *L. buchneri* MVs after 24, 48, and 72 h against AGS and HT-29 cancer cells (P < 0.0001****). Untreated AGS and HT-29 cancer cells were considered as control cells. The results are expressed as mean ± SD of three independent experiments (n = 3). (**C**) Cell viability of HFF normal cells following treatment with *L. buchneri* MVs after 48, and 72 h.

The anticancer effect of MVs isolated from *L. buchneri* against AGS and HT-29 cancer cell lines was investigated at 24, 48, and 72 h time intervals (Fig. 2A), which demonstrated that all concentrations of MVs (12.5, 25, 50, 100, and 200 μg/mL) have the ability to significantly reduce the viability of both types of human adenocarcinoma after 24, 48, and 72 h treatments (P < 0.001). However, 200 μg/mL of MVs showed the highest reduction of cell viability in both cancer cells after 72 h. The IC_50_ of MVs was also calculated against both cancer cells (Fig. 2B), showing that the IC_50_ concentration decreased significantly with increasing time of treatment, and the 72 h treatment shows the lowest calculated IC_50_ against AGS and HT29 cell lines rather than the 24 and 48 h treatment groups (P < 0.001). Figure 2C shows the viability of HFF cells when exposed to different concentrations of MVs. The results indicate that higher concentrations (100 and 200 μg/mL) of MVs significantly reduced the viability of HFF cells after 48 and 72 h of treatment (P < 0.001). A high level of cell viability has been detected in HFF normal cells. More than the 80% cell viability has been reported after 48 and 72 h of treatment with MVs. As shown in the graph, *L. buchneri* MVs exhibited a high level of biocompatibility with normal HFF cells. MVs derived from *L. buchneri* showed a lower level of toxicity against normal HFF cells as compared to HT-29 and AGS cancer cells.

### *L. buchneri* MVs induce apoptosis signaling pathway

**Figure 3:**
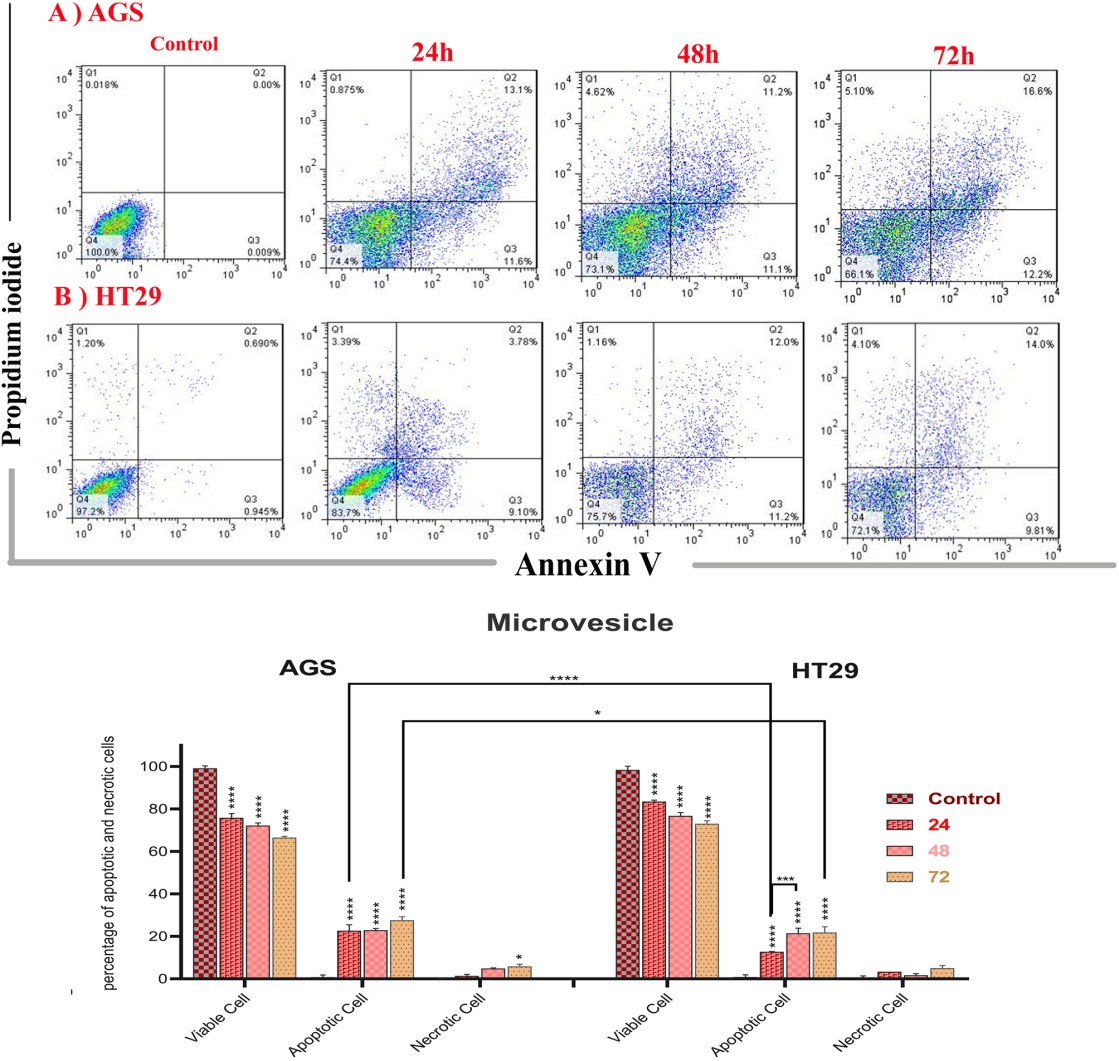
(**A**) Flowcytometric plots to evaluate apoptosis induced by *L. buchneri* MVs at 24, 48, and 72 h in AGS (**A**) and HT-29 (**B**) cancer cells. (P < 0.0001****, P < 0.001***, P < 0.05*). Untreated AGS and HT-29 cells were used as control cells.

A flow cytometry study was used to further investigate cell death (apoptosis or necrosis). Early, late, and necrotic apoptotic cells were stained with Annexin-FITC, PI, and both PI and Annexin-FITC as shown in this section. The percentage of cells in early apoptosis (Q3 region) was 9.10%, 14.3%, 9.81% (HT-29 cells) and 10.9%, 18.6%, 17.2% (AGS cells) after 24, 48, and 72 h of treatment, respectively, and the percentage of cells in late apoptosis (Q2 region) was 3.79%, 7.01%, 14% (HT-29 cells treated for 24, 48, and 72 h, respectively) and 6.05%, 6.97%, 15.7% (AGS cells treated for 24, 48, and 72 h, respectively) (Fig. 3). Analysis of the results also showed that the percentage of AGS cells in early and late apoptotic stages was significantly higher than the control group after 24, 48, and 72 h of treatment (P < 0.001). Also, the percentage of HT-29 cells in early apoptotic stage (in all time interval treatments) and late apoptotic stage (in 48 and 72 h treated groups) was dramatically higher than control group (P < 0.001). Therefore, the apoptosis observed in both AGS and HT-29 cells increased significantly with increasing time of treatment (P < 0.001). The occurrence of apoptosis was higher in AGS cells treated for 24 and 72 h compared to HT-29 cells treated for the same time intervals (P < 0.05 and P < 0.001, respectively).

### Cell cycle arrest analysis

**Figure 4:**
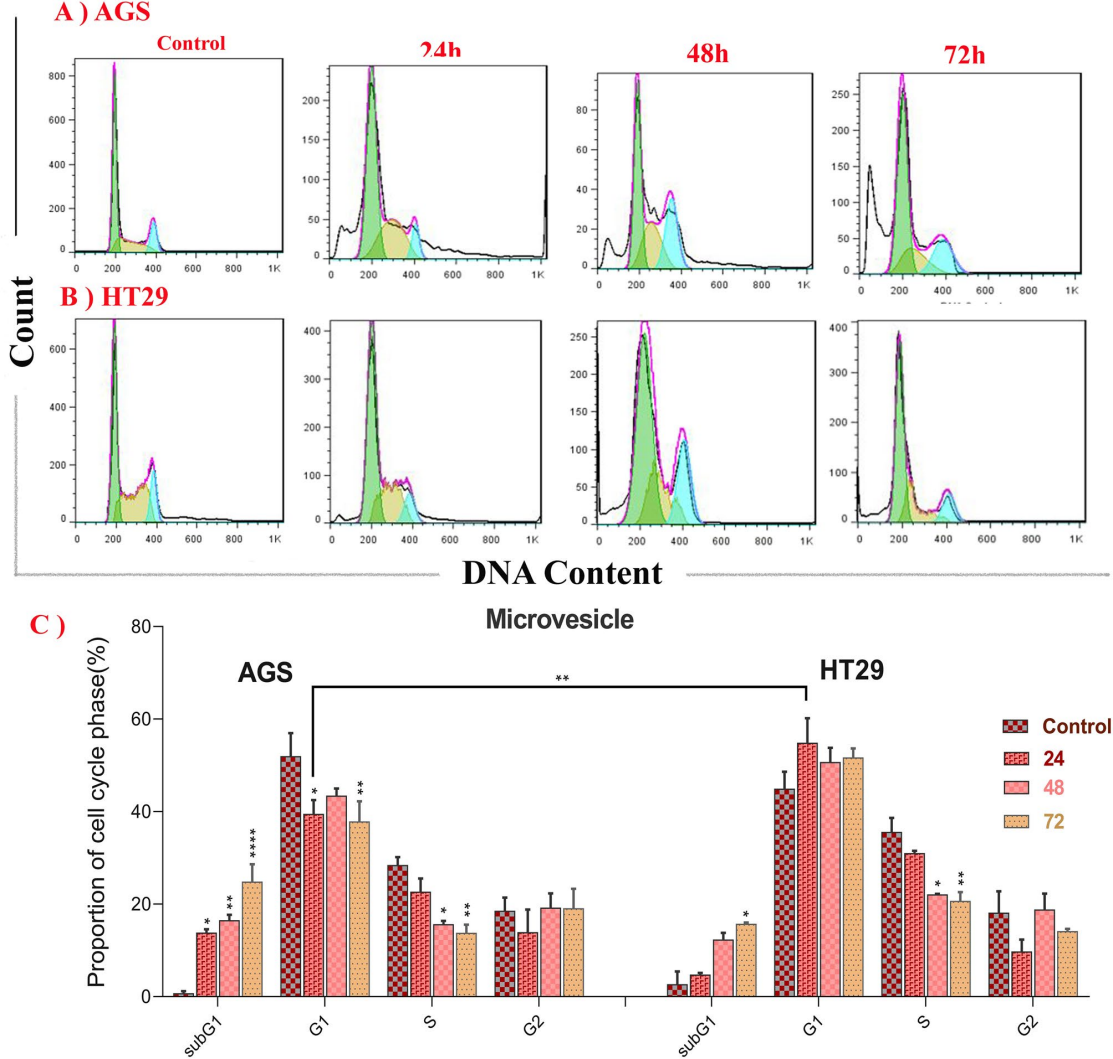
Flow cytometry plots for cell cycle arrest in AGS (**A**) and HT-29 (**B**) cancer cells treated with *L. buchneri* MVs for 24, 48, and 72 h. (P < 0.0001****, P < 0.001***, P < 0.05*). Untreated AGS and HT-29 cells were used as control cells.

After treatment of both AGS and HT-29 cells with MVs, the cells were arrested in the G0/G1 phase (Fig. 4). The percentage of apoptotic cell population was statistically increased in the sub-G1 phase of AGS (after 48 and 72 h of treatment) and HT-29 (after 72 h of treatment) cells compared to the control group. The increase in the sub-G1 phase population of AGS was significantly higher in the 72-h treatment group compared to the other groups (P < 0.05), and also the population of HT-29 cells in sub-G1 and G1 phases was statistically greater than the population of AGS cells in similar phases after 72-h treatment (P < 0.05 and P < 0.0001, respectively). On the other hand, a significant reduction in the population of S phase cells was reported compared to the control group (after 24, 48 and 72 h of treatment for the AGS cell line and after 48 and 72 h of treatment for the HT-29 cell line).

### Evaluation of genes expression

**Figure 5:**
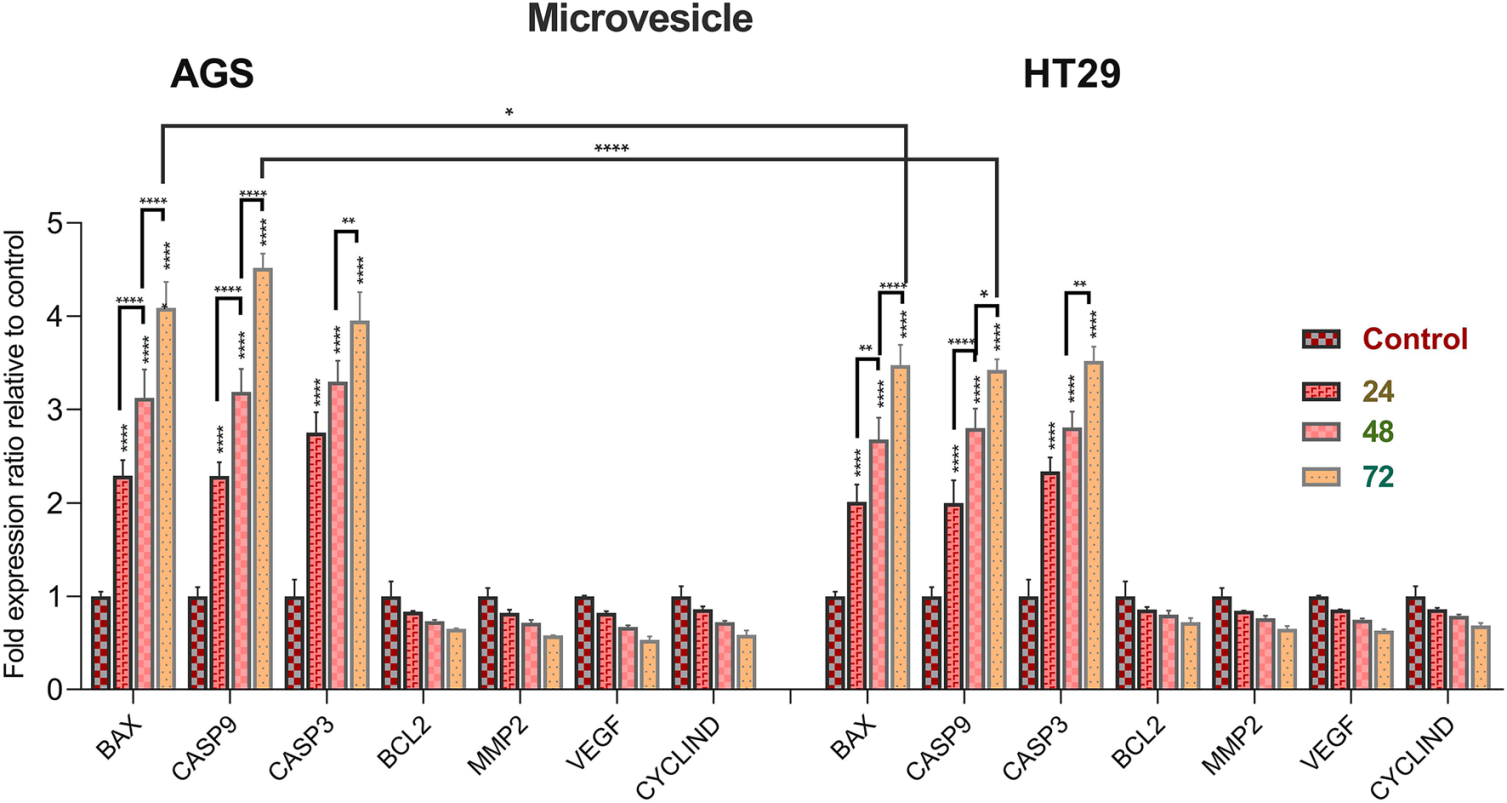
The expression levels of *BAX*,* CASP9*,* CASP3*,* BCL2*,* MMP2*,* VEGF*, and *CCND1* in AGS (**A**) and HT-29 (**B**) cancer cells treated with *L. buchneri* MVs for 24, 48, and 72 h. Untreated AGS and HT-29 cells were used as control cells. Results are reported in comparison with control group (P < 0.0001****, P < 0.01**, P < 0.05*).

The expression of pro-apoptotic, anti-apoptotic, metastatic, regulator of cell cycle progression and angiogenic genes was evaluated in AGS and HT-29 cells treated with MVs for 24, 48 and 72 h (Fig. 5). The results show that the expression of *BAX, CASP3* and *CASP9* genes has significant upregulation in both cell lines for all time intervals treatments (minimum increase was 2.3-fold) (P < 0.001). However, there was no remarkable reduction in the expression of *BCL2*,* MMP2*,* CCND1* and *VEGF* genes after treatments with MVs compared to the control group. Also, the highest increase in expression of proapoptotic genes was reported in both cancer cell lines after 72 h of treatment (P < 0.001). The upregulation of *BAX* and *CASP9* genes in AGS cell line was also dramatically higher than the expression of the same genes in HT-29 cell line after 72 h of treatment, demonstrating that the occurrence of apoptosis was higher in AGS treated cells compared to HT-29 treated cancer cell line (P < 0.05 for *BAX* and P < 0.0001 for *CASP9*).

### The effects of MVs on cell migration

**Figure 6:**
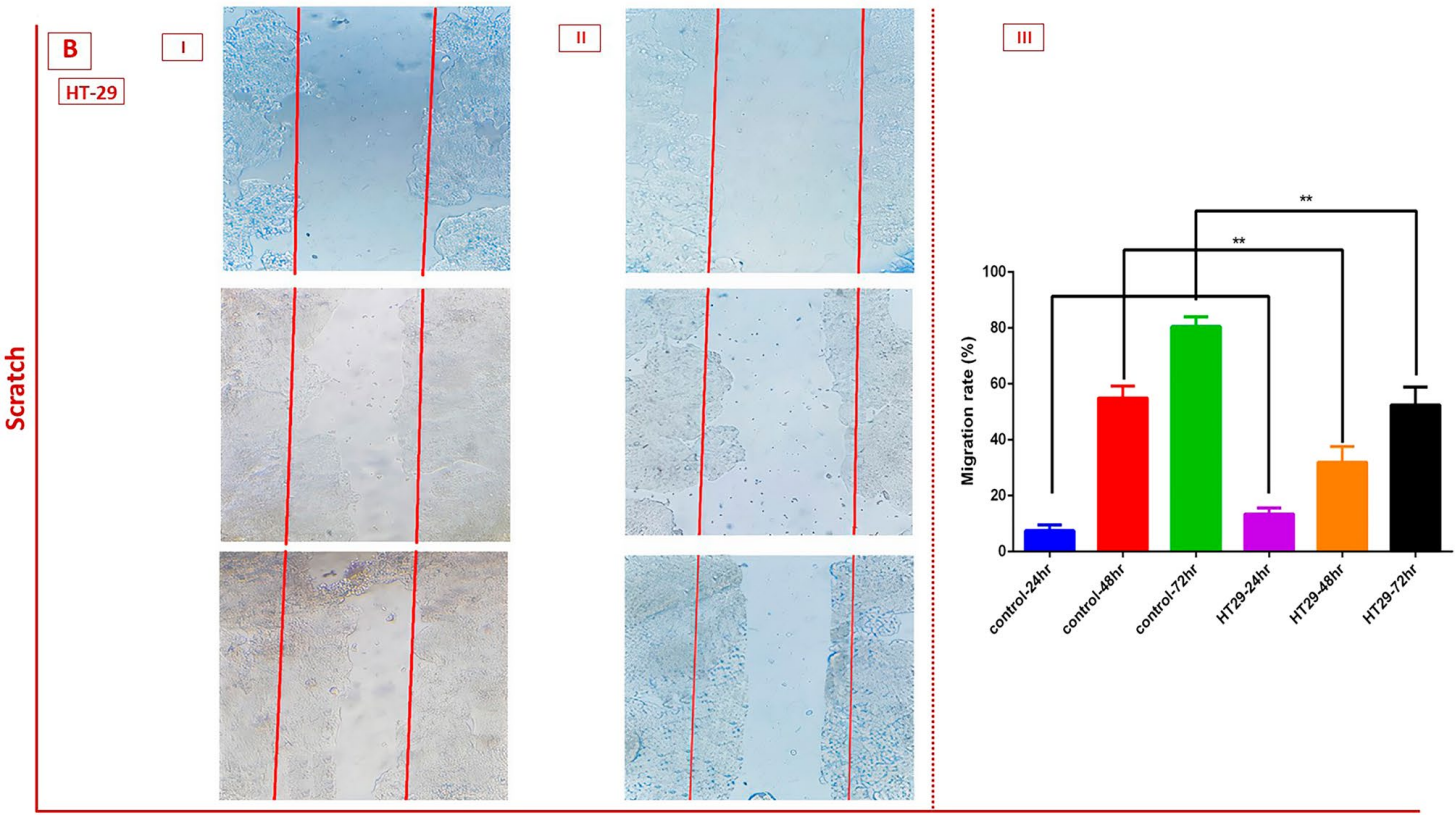
The scratch assay and cell migration ability of (**A**) AGS cells after 24, 48, and 72 h treatment with *L. buchneri* MVs. Graphs showing that the MVs inhibited the migration capabilities of AGS cells (II) after 24, 48, and 72 h treatment compared to control cells. **P < 0.01 and ***P < 0.001. I) Indicates the untreated AGS cancer cells; II) Indicates the treated AGS cancer cells with *L. buchneri* MVs*.* III) Migration rate of cancer cells compare to control cells.

**Figure 7:**
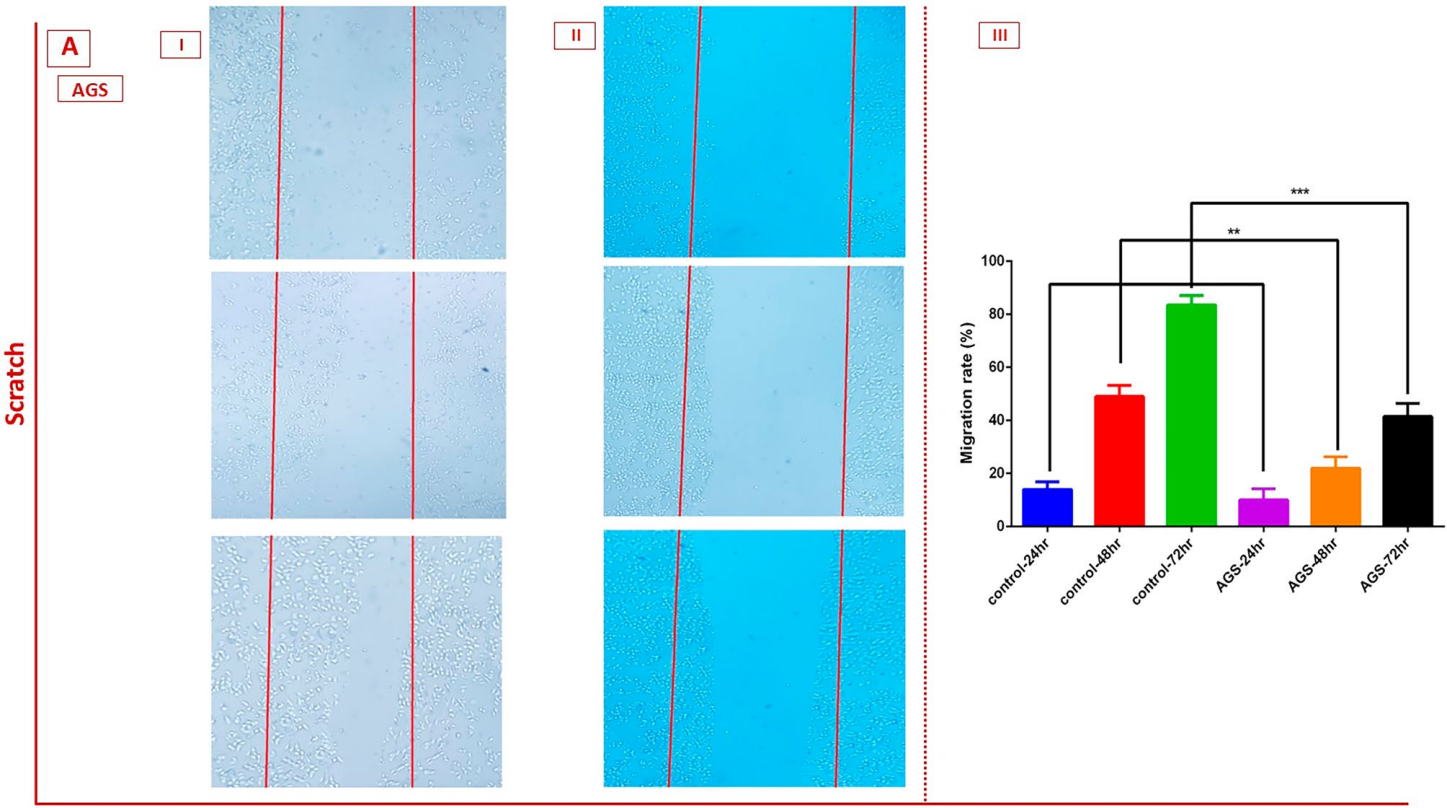
The scratch assay and cell migration ability of (**A**) HT-29 cells after 24, 48, and 72 h treatment with *L. buchneri* MVs. Graphs showing that the MVs inhibited the migration capabilities of HT-29 cells (II) after 24, 48, and 72 h treatment compared to control cells. **P < 0.01 and ***P < 0.001. I) Indicates the untreated HT-29 cancer cells; II) Indicates the treated AGS cancer cells with *L. buchneri* MVs*.* III) Migration rate of cancer cells compare to control cells.

To evaluate the effect of the IC_50_ concentration of MVs on in vitro cell migration, scratch experiments were performed. Over the course of 24, 48, and 72 h of treatment, the movement of treated cells into the denuded region (shown as red lines) was tracked. Figures 6 and 7 show that after 24, 48, and 72 h of incubation in both HT-29 and AGS cancer cell lines, MVs can significantly reduce cell migration compared to the control groups (P < 0.01, P < 0.001).

## Discussion

Probiotics are dietary supplements containing non-pathogenic bacteria that promote the health of the host^38^. Microvesicles, which are extracellular vesicles of the plasma membrane derived from probiotics and range in size from 100 to 1000 nm, are also known as shedding vesicles or ectosomes^39^. Today, various types of encapsulated materials with anticancer properties are introduced and used. Microvesicles are emerging as novel structures for drug delivery and are preferred over conventional encapsulation materials for delivering anticancer drugs due to several advantages. These include improved drug targeting, enhanced drug release, protection of drugs from degradation, biocompatibility and biodegradability, smaller size and improved tissue permeability, and cost-effectiveness. In other words, microvesicles have the potential to be a valuable tool for delivering anticancer drugs. Further research is needed to develop microvesicle-based delivery systems that are safe, effective, and scalable^40–43^.

Studies suggest that microvesicles derived from specific probiotic bacteria, such as *Lactobacillus*, may have immunomodulatory effects and influence processes related to cancer prevention and therapy. Some of the potential mechanisms under investigation include immunomodulation, anti-inflammatory effects, delivery of bioactive molecules, and communication with host cells^44–46^. One novel approach to treating and preventing gastrointestinal cancers is the utilization of human microbiota. Numerous studies have demonstrated a complex relationship between the gut microbiota and cancer progression, as well as its impact on cancer therapy^47^.

Based on these unique features, we chose *L. buchneri* microvesicles to evaluate its anticancer properties. But the reason for choosing this bacterium was also its interesting properties. *L. buchneri*, as a potential probiotic, reduces inflammation and boosts the immune system. Recently, the anticancer properties of this bacterium have also been noticed. This bacterium produces short-chain fatty acids (SCFAs), which have anti-inflammatory properties, and by reducing anti-inflammatory molecules, reduces the risk of cancer development. This anticancer property can be caused by the production of antimicrobial compounds and the simultaneous modulation of the immune system. Before investigating the anticancer properties of microvesicles from this bacterium, it was necessary to first prove the safety and efficacy of the bacterium itself. This evaluation was conducted in accordance with the guidelines of the World Health Organization. For this purpose, after identifying the strain at the molecular level, the pattern of sugar fermentation was determined. The resistance profile to common antibiotics and the presence of antibiotic resistance genes and toxin production were then evaluated. To assess efficiency, the resistance to stomach acid and enzymes, resistance to bile salts, and the ability to attach to CaCO_2_ cell lines were evaluated. This strain was only used in the subsequent tests to evaluate the anticancer effect of microvesicles after its probiotic properties were proven^33,48,49^.

The results of our study show that the measured MVs are less than 200 nm, indicating the accurate size of these MVs. The gel image indicates that the number of proteins in the supernatant is lower than in the cell extract and MVs. Interestingly, one of the reasons may be attributed to the MVs separation process and the ultracentrifuge feature. During this process, a large amount of MVs is separated, showing more proteins compared to the supernatant. Cell wall and secretory proteins mainly consist of intramembrane proteins and enzymes related to the cell wall. Of course, due to the low sensitivity of the SDS-PAGE method, there may be other proteins in the supernatant or MVs that are not visible on the gel. MVs are challenging to differentiate and are frequently used interchangeably due to their similar sizes and associated markers^50^.

For the first time, we found that *L. buchneri* MVs have the potential to effectively inhibit the growth of HT-29 and AGS cancer cell lines. Our findings support the investigators' assertion that the observed changes were both time- and dose-dependent^36^. We have found that these probiotic MVs can be used as an effective anticancer agent with low cytotoxicity against normal cells.

Behzadi et al., demonstrated the inhibitory effects of extracellular vesicles derived from *L. rhamnosus* GG on the growth of hepatic cancer cells^34^. In another study, extracellular vesicles inhibited the growth of Sw480 and HT-29 cells^51^. Inhibitory activity of *L. buchneri* FD2 has been reported against HT-29 cancer cells^52^. Therefore, it is possible that MVs isolated from *L. buchneri* have biological properties that could reduce cancer cell viability and induce apoptotic events in cancer cells. A recent study demonstrated that heat-killed *L. fermentum* KU200060 and *L. buchneri* KU200793 exhibited significant antioxidant activity^53^. The cytotoxic activity of *Lactobacillus* strains against cancer cells may be due to the antioxidant properties of the *Lactobacillus* strains. Several studies have reported that various antioxidant compounds can trigger anticarcinogenic effects against specific types of cancers^52^.

Various studies indicate that *L. rhamnosus* could exert anticancer effects on various gastrointestinal cancer cell lines, including HT-29 and AGS cancer cell lines^54,55^. The results of the research conducted by Dehghani et al. demonstrated that *L. rhamnosus* has the ability to inhibit HT-29 cell growth and induce apoptosis. Depending on the quantity and timing of probiotic bacterial supernatant, it can induce apoptotic induction and anti-proliferative potential. Apoptosis induction was observed at 48 and 72 h compared to the control group, but the stimulation was more pronounced at 48 h^56^. The study by Salemi et al. revealed that the cell-free supernatant of *L. rhamnosus* GG (LGG-SN) specifically and concentration-dependently reduces the survival of cancer cells. For cancer cell lines, the relative IC_50_ values vary from 91.8% LGG-SN (v/v) for the less sensitive Caco-2 cells to 73.8% LGG-SN (v/v) for HT-29 and 74.8% LGG-SN (v/v) for A375, respectively^57^. According to the MTT analysis of Vachkova et al. *Lacticaseibacillus paracasei* strains can inhibit the growth of HT-29 cells in a dose-dependent manner^58^.

Additionally, no high level of cytotoxicity was observed against normal HFF cells, which is consistent with other studies^59–61^. In our study, the cytotoxicity results of *L. buchneri* showed selective inhibition of cancer cells in comparison to normal cells. In another study, *L. buchneri* FD2 showed selectivity in killing cancer cells compared to the WRL68 normal liver cell line. In addition, research has shown that *Lactiplantibacillus plantarum* YT013 and the cell-free culture supernatant from serofluid dish could inhibit the proliferation of the cancer cell lines without causing harm to healthy cells^62^.

According to our research, *L. buchneri* MVs induced apoptosis in both the HT-29 and AGS cell lines in vitro and can suppress the growth of cancer cells. Several probiotics and the compounds they produce have also been shown to have the ability to inhibit the growth and induce apoptosis of cancer cells^63,64^. Apoptosis, a form of regulated cell death, is essential for growth and tissue homeostasis. Cancer cell growth can be controlled by triggering the process of apoptosis. Most anticancer drugs trigger apoptosis. *Lactobacillus* species can induce apoptosis in cancer cells and inhibit their proliferation by regulating the expression of specific BCL-2 family proteins, producing reactive oxygen species (ROS), inducing the translocation of calreticulin (CRT), and altering the cell cycle, ultimately leading to cell death^65,66^. Certain bacterial extracellular vesicles have been shown to bind selectively to the surface of the host cell^67,68^.

The researchers confirmed that extracellular vesicles (EVs) can be taken up by colon cancer cells and inhibit their growth by inducing apoptosis^69^. Extracellular vesicles produced by *L. paracasei* (LpEVs) have been demonstrated to reduce colorectal cancer cell proliferation and induce apoptosis through the PDK1/AKT/Bcl-2 pathway^70^. EVs from *L. rhamnosus* GG prevent the growth of liver cancer cells by inducing apoptosis. *L. rhamnosus* GG EVs can increase the apoptotic index (*BAX/BCL2* expression ratio) in liver cancer cells in a dose-dependent manner^34^. Previous research has indicated that EVs from *L. rhamnosus* GG exhibited anticancer properties on the HepG2 cell line^71^.

*L. acidophilus* CICC 6074 has been shown to be capable of reversing the apoptotic process in HT-29 cells. The production of the anti-apoptotic protein BCL2 may be inhibited by *L. acidophilus* CICC 6074, which in turn may lead to a decrease in the capacity of the mitochondrial membrane^72^.

The results of qRT-PCR showed that the treatment of cancer cells with *L. buchneri* MVs led to a remarkable enhancement in the expression of *BAX*,* CASP3*, and *CASP9* genes. Additionally, the apoptotic index (*BAX/BCL2* expression ratio) increased significantly in our study. The elevated BAX/BCL2 ratio following treatment with *L. rhamnosus* GG extracellular vesicles was associated with apoptosis in liver cancer^34^.

A broad family of proteins known as BCL2 regulates both cell death and cell survival. The BCL2 family of proteins regulates the structural integrity of the mitochondrial outer membrane and is crucial for the mitochondria-mediated apoptotic process. In this study, the anti-apoptotic protein BCL2 and the pro-apoptotic protein BAX, both members of the BCL2 family, were investigated. Increased BAX expression appeared to activate the intrinsic mitochondrial pathway. This led to an increase in caspase-3 and caspase-9, the release of cytochrome c, and the onset of apoptosis^73^.

To prevent the transfer of BAX to the mitochondrial membrane, BCL2 binds to it. Thus, it is predicted that the *BAX* gene would increase and the *BCL2* gene would decrease in an apoptotic cell. Therefore, the ratio of BAX to BCL2 could be used to predict the behavior of the cells in the apoptotic system^74^. The probiotic supernatant degrades the integrity of chromatin within the nucleus by increasing reactive oxygen species and lipid peroxidation, as well as decreasing mitochondrial membrane potential, increasing caspase-3 activity, and decreasing BCL2 anti-apoptotic activity^75^.

No significant downregulation in the expression of *MMP2*,* CCND1*, and *VEGF* genes was observed following treatment with *L. buchneri* MVs. Numerous studies indicate that this probiotic and its metabolites have a high ability to decrease the expression of the mentioned genes. In a previous study, it was found that treating colorectal cancer cells with *L. rhamnosus* GG extracellular vesicles could significantly reduce the mRNA expression of the MMP2 and MMP9 genes^71^. As a result, targeted destruction of *CCND1* may be an interesting target for cancer treatment^76^. After 48 and 72 h of treatment with heat-killed *L. brevis* and *L. paracasei*, HT-29 cells showed a decrease in *BCL2* mRNA expression compared to untreated cells^77^. After treatment with *L. rhamnosus*, the expression of the anti-apoptotic *BCL2* genes and genes required for cell cycle progression (*CCND1* and *CCNE*) decreased in HT-29 cells^56^.

We also investigated the impact of the microvesicles on cancer cell adherence and the expression of MMP2, a gene involved in cancer development, invasion, and metastasis. It has been reported that MMP2 is also upregulated in various solid and hematologic malignancies, thereby promoting cancer development^78^. As one of the major angiogenic factors in tumors, vascular endothelial growth factor (VEGF) plays a crucial role in the early stages of tumor growth, progression, and metastasis. As a result, one of the most important therapeutic targets for the treatment of many malignancies is VEGF and the receptor-mediated signaling pathways it activates^79^.

In addition, studies have shown that metabolites secreted by *L. plantarum* YYC-3 can suppress the VEGF-MMP2/9 signaling pathway, thereby preventing metastasis of colon cancer cells^80^. Further research is needed to understand the additional mechanisms by which *L. buchneri* MVs exert apoptotic effects by altering the expression of pro-apoptotic, anti-apoptotic, metastatic, and angiogenic cancer-related genes.

The cell cycle analysis in our study revealed that MVs could lead to a significant increase in the sub-G1 phase and a remarkable decrease in the S phase in the tested cancer cells, in a time-dependent manner. Consistent with our results, a cell cycle investigation in another study demonstrated that in the HT-29 cancer cell line, *L. rhamnosus* supernatant increased the peak of sub-G1 and induced cell cycle arrest in the G0/G1 phase^56^. According to research, *L. rhamnosus* GG and *Bifidobacterium lactis* Bb12 supernatant can induce apoptosis by reducing BCL2, regulating BAK, slowing the growth of HT-29 cells, and stopping cell division by arresting cells in the G0/G1 phase^81^. Reports on the impact of probiotic MVs on cancer cell cycle arrest are limited.

In the current study, we also observed that the migration ability of cancer cells was suppressed during treatment with MVs from a specific probiotic strain. This finding is consistent with other research indicating that probiotics and their metabolites may inhibit the progression of various cancers by interfering with migration and invasion processes^82–84^.

## Conclusion

**Figure 8:**
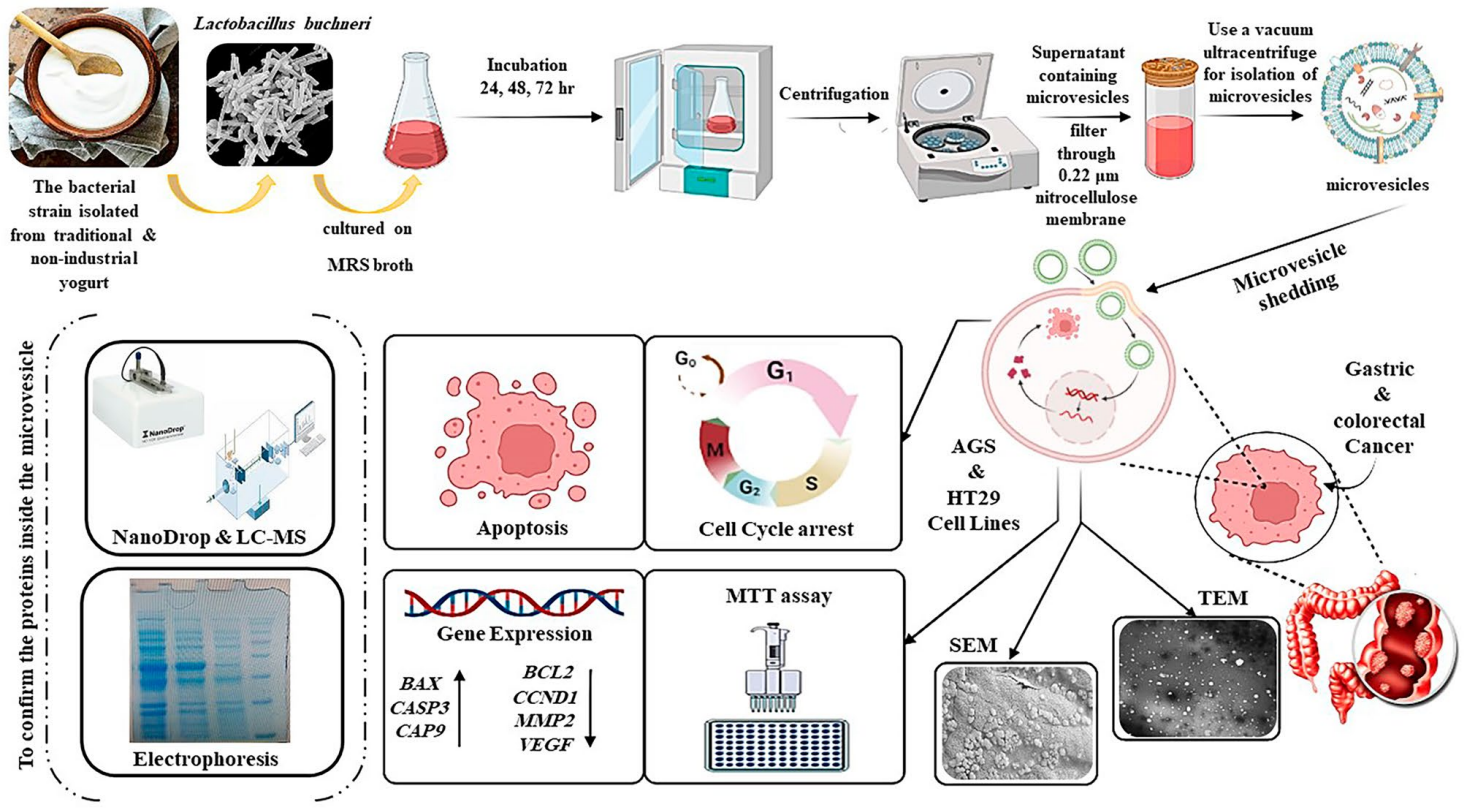
The impacts of *L. buchneri* MVs in apoptosis induction and cell cycle arrest on AGS gastric cancer and HT-29 colon cancer cells.

The metabolites of probiotic bacteria may affect human health. They appear to be a promising treatment option for cancer. To the best of our knowledge, there have been no studies investigating the anticancer and apoptotic properties of *L. buchneri* probiotic. The anticancer activity of *L. buchneri* microvesicles was assessed against gastric and colon cancer cell lines, demonstrating the dose- and time-dependent apoptotic effects of these extracellular vesicles. These microvesicles demonstrate apoptotic activity by upregulating pro-apoptotic genes and inducing cell cycle arrest in the sub-G1 phase. The microvesicles of *L. buchneri* can also be considered as a potential option to inhibit the activities of cancer cells. The microvesicles of *L. buchneri* can also be considered as a potential option for cancer therapy (Fig. 8).

### Supplementary Information

Supplementary Information 1.

## Data Availability

The datasets used and/or analyzed during the current study are available from the corresponding author on reasonable request.

## References

[1] Zendeboodi F, Khorshidian N, Mortazavian AM, da Cruz AG (2020). Probiotic: Conceptualization from a new approach. Curr. Opin. Food Sci..

[2] Bermudez-Brito M, Plaza-Díaz J, Muñoz-Quezada S, Gómez-Llorente C, Gil A (2012). Probiotic mechanisms of action. Ann. Nutr. Metab..

[3] Cremon C, Barbaro MR, Ventura M, Barbara G (2018). Pre-and probiotic overview. Curr. Opin. Pharmacol..

[4] Tamime AY, Saarela M, Sondergaard AK, Mistry V, Shah N (2005). Production and maintenance of viability of probiotic microorganisms in dairy products. Probiot. Dairy Prod..

[5] Haghshenas B, Kiani A, Nami Y (2021). Probiotic potential and safety evaluation of lactic acid bacteria isolated from colostrum. J. Biosaf..

[6] Moayyedi P, Ford AC, Talley NJ, Cremonini F, Foxx-orenstein A, Brandt L, Quigley E (2008). The efficacy of probiotics in the therapy of irritable bowel syndrome: A systematic review. Gut.

[7] Gourbeyre P, Denery S, Bodinier M (2011). Probiotics, prebiotics, and synbiotics: Impact on the gut immune system and allergic reactions. J. Leukoc. Biol..

[8] Frick J-S, Schenk K, Quitadamo M, Kahl F, Köberle M, Bohn E (2007). Lactobacillus fermentum attenuates the proinflammatory effect of *Yersinia enterocolitica* on human epithelial cells. Inflamm. Bowel Dis..

[9] Macpherson AJ, Harris NL (2004). Interactions between commensal intestinal bacteria and the immune system. Nat. Rev. Immunol..

[10] Rafter J, Bennett M, Caderni G, Clune Y, Hughes R, Karlsson PC (2007). Dietary synbiotics reduce cancer risk factors in polypectomized and colon cancer patients. Am. J. Clin. Nutr..

[11] Ferdousi R, Rouhi M, Mohammadi R, Mortazavian AM, Khosravi-Darani K, Rad AH (2013). Evaluation of probiotic survivability in yogurt exposed to cold chain interruption. Iran. J. Pharm. Res..

[12] Kiani A, Nami Y, Hedayati S, Jaymand M, Samadian H, Haghshenas B (2021). Tarkhineh as a new microencapsulation matrix improves the quality and sensory characteristics of probiotic *Lactococcus lactis* KUMS-T18 enriched potato chips. Sci. Rep..

[13] Haghshenas B, Nami Y, Kiani A, Moazami N, Tavallaei O (2023). Cytotoxic effect of potential probiotic *Lactiplantibacillus plantarum* KUMS-Y8 isolated from traditional dairy samples on the KB and OSCC human cancer cell lines. Heliyon.

[14] Adolfsson O, Meydani SN, Russell RM (2004). Yogurt and gut function. Am. J. Clin. Nutr..

[15] Willett, W. C., Koplan, J. P., Nugent, R., Dusenbury, C., Puska, P., & Gaziano, T. A. Prevention of chronic disease by means of diet and lifestyle changes. Disease Control Priorities in Developing Countries 2nd edition (2006). 21250366

[16] Ayivi RD, Gyawali R, Krastanov A, Aljaloud SO, Worku M, Tahergorabi R (2020). Lactic acid bacteria: Food safety and human health applications. Dairy.

[17] Goodman B, Gardner H (2018). The microbiome and cancer. J. Pathol..

[18] Markowiak P, Śliżewska K (2018). The role of probiotics, prebiotics and synbiotics in animal nutrition. Gut Pathog..

[19] Ağagündüz D, Yılmaz B, Şahin TÖ, Güneşliol BE, Ayten Ş, Russo P (2021). Dairy lactic acid bacteria and their potential function in dietetics: The food–gut-health axis. Foods.

[20] Beev G, Michaylova M, Dinev T, Naydenova N, Tzanova M, Urshev Z (2021). ARDRA Analysis on biodiversity of lactobacilli isolated from Bulgarian raw buffalo milk. Acta Microbiol. Bulg..

[21] Benavides AB, Ulcuango M, Yépez L, Tenea GN (2016). Assessment of the in vitro bioactive properties of lactic acid bacteria isolated from native ecological niches of Ecuador. Rev. Argent. Microbiol..

[22] Cano-Garrido O, Seras-Franzoso J, Garcia-Fruitós E (2015). Lactic acid bacteria: Reviewing the potential of a promising delivery live vector for biomedical purposes. Microbial Cell Factor..

[23] Wang M, Gao Z, Zhang Y, Pan L (2016). Lactic acid bacteria as mucosal delivery vehicles: A realistic therapeutic option. Appl. Microbial. Biotechnol..

[24] Kuczkowska K, Kleiveland CR, Minic R, Moen LF, Øverland L, Tjåland R (2017). Immunogenic properties of *Lactobacillus plantarum* producing surface-displayed *Mycobacterium tuberculosis* antigens. Appl. Environ. Microbiol..

[25] Deatherage BL, Cookson BT (2012). Membrane vesicle release in bacteria, eukaryotes, and archaea: A conserved yet underappreciated aspect of microbial life. Infect. Immunity.

[26] Pathirana RD, Kaparakis-Liaskos M (2016). Bacterial membrane vesicles: Biogenesis, immune regulation and pathogenesis. Cell. Microbiol..

[27] Domínguez Rubio AP, Martínez JH, Martínez Casillas DC, Coluccio Leskow F, Piuri M, Pérez OE (2017). *Lactobacillus casei* BL23 produces microvesicles carrying proteins that have been associated with its probiotic effect. Front. Microbiol..

[28] Brown L, Kessler A, Cabezas-Sanchez P, Luque-Garcia JL, Casadevall A (2014). Extracellular vesicles produced by the G ram-positive bacterium *B. acillus* subtilis are disrupted by the lipopeptide surfactin. Mol. Microbiol..

[29] Li M, Lee K, Hsu M, Nau G, Mylonakis E, Ramratnam B (2017). Lactobacillus-derived extracellular vesicles enhance host immune responses against vancomycin-resistant enterococci. BMC Microbiol..

[30] Al-Nedawi K, Mian MF, Hossain N, Karimi K, Mao YK, Forsythe P (2015). Gut commensal microvesicles reproduce parent bacterial signals to host immune and enteric nervous systems. FASEB J..

[31] Hashemi-Khah M-S, Arbab-Soleimani N, Forghanifard M-M, Gholami O, Taheri S, Amoueian S (2022). An In vivo study of *Lactobacillus rhamnosus* (PTCC 1637) as a new therapeutic candidate in esophageal cancer. BioMed Res. Int..

[32] Nami Y, Abdullah N, Haghshenas B, Radiah D, Rosli R, Khosroushahi AY (2014). Assessment of probiotic potential and anticancer activity of newly isolated vaginal bacterium *Lactobacillus plantarum* 5BL. Microbiol. Immunol..

[33] Abedi A, Tafvizi F, Akbari N, Jafari P (2023). Cell-free supernatant of *L. buchneri* probiotic bacteria enhancing apoptosis activity in AGS gastric cancer cells. Iran. J. Sci..

[34] Behzadi E, Hosseini HM, Fooladi AAI (2017). The inhibitory impacts of *Lactobacillus rhamnosus* GG-derived extracellular vesicles on the growth of hepatic cancer cells. Microbial Pathog..

[35] Ghaderi F, Sotoodehnejadnematalahi F, Hajebrahimi Z, Fateh A, Siadat SD (2022). Effects of active, inactive, and derivatives of *Akkermansia muciniphila* on the expression of the endocannabinoid system and PPARs genes. Sci. Rep..

[36] Dolati M, Tafvizi F, Salehipour M, Movahed TK, Jafari P (2021). Inhibitory effects of probiotic *Bacillus coagulans* against MCF7 breast cancer cells. Iran. J. Microbiol..

[37] Haji Mehdi Nouri Z, Tafvizi F, Amini K, Khandandezfully N, Kheirkhah B (2023). Enhanced induction of apoptosis and cell cycle arrest in MCF-7 breast cancer and HT-29 colon cancer cell lines via low-dose biosynthesis of selenium nanoparticles utilizing *Lactobacillus casei*. Biol. Trace Elem. Res..

[38] Lu K, Dong S, Wu X, Jin R, Chen H (2021). Probiotics in cancer. Front. Oncol..

[39] Katsuda T, Kosaka N, Ochiya T (2014). The roles of extracellular vesicles in cancer biology: Toward the development of novel cancer biomarkers. Proteomics.

[40] Chen L, Wang L, Zhu L, Xu Z, Liu Y, Li Z (2022). Exosomes as drug carriers in anti-cancer therapy. Front. Cell Dev. Biol..

[41] Wang Z, Mo H, He Z, Chen A, Cheng P (2022). Extracellular vesicles as an emerging drug delivery system for cancer treatment: Current strategies and recent advances. Biomed. Pharmacother..

[42] Wu M, Wang M, Jia H, Wu P (2022). Extracellular vesicles: Emerging anti-cancer drugs and advanced functionalization platforms for cancer therapy. Drug Deliv..

[43] Zhang X, Zhang H, Gu J, Zhang J, Shi H, Qian H (2021). Engineered extracellular vesicles for cancer therapy. Adv. Mater..

[44] González-Lozano E, García-García J, Gálvez J, Hidalgo-García L, Rodríguez-Nogales A, Rodríguez-Cabezas ME, Sánchez M (2022). Novel horizons in postbiotics: Lactobacillaceae extracellular vesicles and their applications in health and disease. Nutrients.

[45] Hosseini-Giv N, Basas A, Hicks C, El-Omar E, El-Assaad F, Hosseini-Beheshti E (2022). Bacterial extracellular vesicles and their novel therapeutic applications in health and cancer. Front. Cell. Infect. Microbiol..

[46] Amatya SB, Salmi S, Kainulainen V, Karihtala P, Reunanen J (2021). Bacterial extracellular vesicles in gastrointestinal tract cancer: An unexplored territory. Cancers.

[47] Raskov H, Burcharth J, Pommergaard H-C (2017). Linking gut microbiota to colorectal cancer. J. Cancer.

[48] Ramakrishna, B., Nair, G., & Takeda, Y. FAO/WHO guidelines to assess probiotic efficacy for human consumption–would they suffice. ECAB health impact of probiotics: Vision & opportunities-E-Book. 2014;105.

[49] Renchinkhand G, Magsar U, Bae HC, Choi S-H, Nam MS (2022). Identification of β-glucosidase activity of *Lentilactobacillus buchneri* URN103L and its potential to convert ginsenoside Rb1 from Panax ginseng. Foods.

[50] Lim W, Kim H-S (2019). Exosomes as therapeutic vehicles for cancer. Tissue Eng. Regener. Med..

[51] Keyhani G, Hosseini HM, Salimi A (2022). Effect of extracellular vesicles of *Lactobacillus rhamnosus* GG on the expression of CEA gene and protein released by colorectal cancer cells. Iran. J. Microbiol..

[52] Shokryazdan P, Jahromi M, Liang J, Sieo C, Kalavathy R, Idrus Z, Ho Y (2017). In vitro assessment of bioactivities of Lactobacillus strains as potential probiotics for humans and chickens. J. Food Sci..

[53] Cheon M-J, Lim S-M, Lee N-K, Paik H-D (2020). Probiotic properties and neuroprotective effects of *Lactobacillus buchneri* KU200793 isolated from Korean fermented foods. Int. J. Mol. Sci..

[54] Sivamaruthi BS, Kesika P, Chaiyasut C (2020). The role of probiotics in colorectal cancer management. Evid. Based Complement. Altern. Med..

[55] Rahimi AM, Nabavizadeh F, Ashabi G, Halimi S, Rahimpour M, Vahedian J, Panahi M (2021). Probiotic *Lactobacillus rhamnosus* supplementation improved capecitabine protective effect against gastric cancer growth in male BALB/c mice. Nutr. Cancer.

[56] Dehghani N, Tafvizi F, Jafari P (2021). Cell cycle arrest and anti-cancer potential of probiotic *Lactobacillus rhamnosus* against HT-29 cancer cells. BioImpacts.

[57] Salemi R, Vivarelli S, Ricci D, Scillato M, Santagati M, Gattuso G (2023). *Lactobacillus rhamnosus* GG cell-free supernatant as a novel anti-cancer adjuvant. J. Transl. Med..

[58] Vachkova E, Petrova V, Grigorova N, Ivanova Z, Beev G (2023). Evaluation of the anticancer and probiotic potential of autochthonous (Wild) *Lacticaseibacillus paracasei* strains from new ecological niches as a possible additive for functional dairy foods. Foods.

[59] Motevaseli E, Shirzad M, Akrami SM, Mousavi A-S, Mirsalehian A, Modarressi MH (2013). Normal and tumour cervical cells respond differently to vaginal lactobacilli, independent of pH and lactate. J. Med. Microbiol..

[60] Chuah L-O, Foo HL, Loh TC, Mohammed Alitheen NB, Yeap SK, Abdul Mutalib NE (2019). Postbiotic metabolites produced by *Lactobacillus plantarum* strains exert selective cytotoxicity effects on cancer cells. BMC Complement. Altern. Med..

[61] Salek F, Mirzaei H, Khandaghi J, Javadi A, Nami Y (2023). Apoptosis induction in cancer cell lines and anti-inflammatory and anti-pathogenic properties of proteinaceous metabolites secreted from potential probiotic *Enterococcus faecalis* KUMS-T48. Sci. Rep..

[62] Zhang R, Zhou Z, Ma Y, Du K, Sun M, Zhang H (2022). Anti-gastric cancer activity of the cell-free culture supernatant of serofluid dish and *Lactiplantibacillus plantarum* YT013. Front. Bioeng. Biotechnol..

[63] Rosa LS, Santos ML, Abreu JP, Balthazar CF, Rocha RS, Silva HL (2020). Antiproliferative and apoptotic effects of probiotic whey dairy beverages in human prostate cell lines. Food Res. Int..

[64] Konishi H, Fujiya M, Tanaka H, Ueno N, Moriichi K, Sasajima J (2016). Probiotic-derived ferrichrome inhibits colon cancer progression via JNK-mediated apoptosis. Nat. Commun..

[65] Isazadeh A, Hajazimian S, Shadman B, Safaei S, Bedoustani AB, Chavoshi R (2020). Anti-cancer effects of probiotic lactobacillus acidophilus for colorectal cancer cell line caco-2 through apoptosis induction. Pharm. Sci..

[66] Maleki-Kakelar H, Dehghani J, Barzegari A, Barar J, Shirmohamadi M, Sadeghi J, Omidi Y (2020). *Lactobacillus plantarum* induces apoptosis in gastric cancer cells via modulation of signaling pathways in *Helicobacter pylori*. BioImpacts.

[67] Kim OY, Park HT, Dinh NTH, Choi SJ, Lee J, Kim JH (2017). Bacterial outer membrane vesicles suppress tumor by interferon-γ-mediated antitumor response. Nat. Commun..

[68] Engevik MA, Danhof HA, Ruan W, Engevik AC, Chang-Graham AL, Engevik KA (2021). *Fusobacterium nucleatum* secretes outer membrane vesicles and promotes intestinal inflammation. MBio.

[69] Krzyżek P, Marinacci B, Vitale I, Grande R (2023). Extracellular vesicles of probiotics: Shedding light on the biological activity and future applications. Pharmaceutics.

[70] Shi Y, Meng L, Zhang C, Zhang F, Fang Y (2022). Extracellular vesicles of *Lacticaseibacillus paracasei* PC-H1 induce colorectal cancer cells apoptosis via PDK1/AKT/Bcl-2 signaling pathway. Microbiol. Res..

[71] Parsa AM, Mahmoodzadeh Hosseini H, Mirhosseini SA (2022). Influence of extracellular vesicles from *Lactobacillus rhamnosus* GG on the cell adhesion and mmp2 and mmp9 genes expression in colorectal cancer cells. J. Appl. Biotechnol. Rep..

[72] Guo Y, Zhang T, Gao J, Jiang X, Tao M, Zeng X (2020). *Lactobacillus acidophilus* CICC 6074 inhibits growth and induces apoptosis in colorectal cancer cells in vitro and in HT-29 cells induced-mouse model. J. Funct. Foods.

[73] Tukenmez U, Aktas B, Aslim B, Yavuz S (2019). The relationship between the structural characteristics of lactobacilli-EPS and its ability to induce apoptosis in colon cancer cells in vitro. Sci. Rep..

[74] Fan T-J, Han L-H, Cong R-S, Liang J (2005). Caspase family proteases and apoptosis. Acta Biochim. Biophys. Sin..

[75] Aminger W, Hough S, Roberts SA, Meier V, Spina AD, Pajela H (2021). Preservice secondary science teachers’ implementation of an NGSS practice: Using mathematics and computational thinking. J. Sci. Teacher Educ..

[76] Chen S, Li L (2022). Degradation strategy of cyclin D1 in cancer cells and the potential clinical application. Front. Oncol..

[77] Karimi Ardestani S, Tafvizi F, Tajabadi EM (2019). Heat-killed probiotic bacteria induce apoptosis of HT-29 human colon adenocarcinoma cell line via the regulation of Bax/Bcl2 and caspases pathway. Human Exp. Toxicol..

[78] Koch J, Mönch D, Maaß A, Mangold A, Gužvić M, Mürdter T (2022). Pharmacologic targeting of Mmp2/9 decreases peritoneal metastasis formation of colorectal cancer in a human ex vivo peritoneum culture model. Cancers.

[79] Yang Y, Cao Y (2022). The impact of VEGF on cancer metastasis and systemic disease. Semin. Cancer Biol..

[80] Yue Y-C, Yang B-Y, Lu J, Zhang S-W, Liu L, Nassar K (2020). Metabolite secretions of *Lactobacillus plantarum *YYC-3 may inhibit colon cancer cell metastasis by suppressing the VEGF-MMP2/9 signaling pathway. Microbial Cell Factor..

[81] Borowicki A, Michelmann A, Stein K, Scharlau D, Scheu K, Obst U, Glei M (2011). Fermented wheat aleurone enriched with probiotic strains LGG and Bb12 modulates markers of tumor progression in human colon cells. Nutr. Cancer.

[82] Costanzo M, Cesi V, Palone F, Pierdomenico M, Colantoni E, Leter B (2018). Krill oil, vitamin D and *Lactobacillus reuteri* cooperate to reduce gut inflammation. Beneficial Microbes..

[83] Shang F, Jiang X, Wang H, Chen S, Wang X, Liu Y (2020). The inhibitory effects of probiotics on colon cancer cells: In vitro and in vivo studies. J. Gastrointest. Oncol..

[84] Dallal MMS, Mojarrad M, Baghbani F, Raoofian R, Mardaneh J, Salehipour Z (2015). Effects of probiotic *Lactobacillus acidophilus* and *Lactobacillus casei* on colorectal tumor cells activity (CaCo-2). Arch. Iran. Med..

