# Supplementary figures and images for: The inhibition effects of Lentilactobacillus buchneri-derived membrane vesicles on AGS and HT-29 cancer cells by inducing cell apoptosis
Source: Sci Rep. 2024 Feb 7;14:3100. doi: 10.1038/s41598-024-53773-y (PMC10850327; doi:10.1038/s41598-024-53773-y)

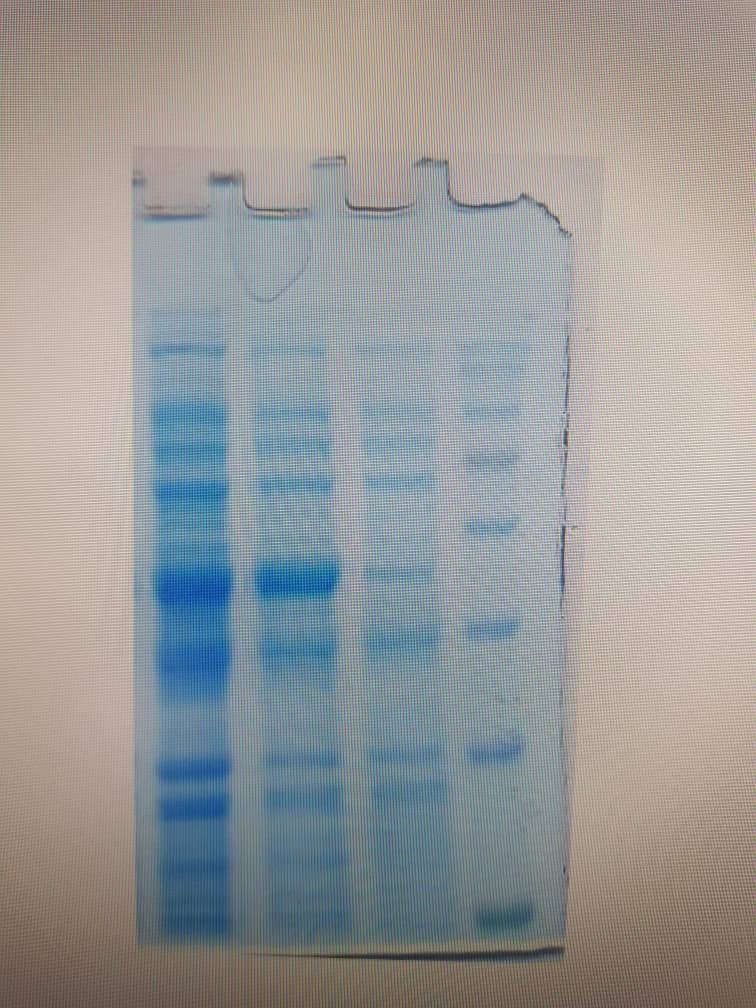

Supplement: Supplementary file 1 — Supplementary Information 1. [file 41598_2024_53773_MOESM1_ESM.jpeg]
